# A review of on-farm recording tools for smallholder dairy farming in developing countries

**DOI:** 10.1007/s11250-024-04024-9

**Published:** 2024-05-20

**Authors:** Yuni Resti, Gustavo Gutierrez Reynoso, Lorenz Probst, Sofiyanti Indriasari, Gema Parasti Mindara, Annisa Hakim, Maria Wurzinger

**Affiliations:** 1 Institute of Livestock Sciences, Department of Sustainable Agricultural Systems, BOKU University, Vienna, Austria; 2 Faculty of Animal Science, Universidad Nacional Agraria La Molina, Lima, Peru; 3 Institute of Development Research, Department of Sustainable Agricultural Systems, BOKU University, Vienna, Austria; 4 Faculty of Computer Science, University of Indonesia, Depok, Indonesia; 5 College of Vocational Studies, IPB University, Bogor, Indonesia

**Keywords:** Developing countries, Smallholder dairy farmers, Dairy recording system, Dairy recording tools

## Abstract

The dairy industry has been expanding significantly recently, which has prompted the improvement and adoption of increasingly digital dairy recording tools with cutting-edge technology. The study aimed to identify smallholder dairy farmers’ recording tools in developing countries. The study presents the results of an extensive literature review conducted using electronic journal databases. The review involved applying a combination of search terms and Boolean operators. The search found a total of 412 research publications. However, only 21 articles with 24 recording tools were deemed appropriate and were subsequently included in the study. Dairy recording entails gathering and managing data on animal information, traceability, health, and productivity that can be conducted using various methods, from manual record-keeping to digitization. The results show that most studies have endeavoured to develop digital recording tools that focus on production performance (PR), mainly milk production, using the Internet of Things (IoT) and mobile phone applications. Moreover, various technologies, such as networks, desktops, and web apps, have also been invented. Given the widespread ownership of mobile phones among the general population, the use of mobile phones continues to be an appealing choice for recording tools. To enhance the advancement of these tools, it is necessary to address technological obstacles, particularly those associated with access and connectivity. In addition, it is also important to consider the continuity of data input and feedback obtained to the farmers, thus helping them evaluate their farms periodically.

## Introduction

The United Nations estimates that there will be 8.5 billion people on the planet by 2030, contributing to the rising demand for food and impacting the growth of the livestock industry, particularly the dairy sector (United Nations 2015). Cattle contribute almost 80% of milk production globally. Other dairy animals such as buffalos, goats, sheep, and camels produce only a tiny proportion (Arrichiello et al. 2022). Due to rising demand in Asia, as well as in Africa and Latin America, it is predicted that the worldwide milk market will expand by 35% in 2030, mostly for India and China (Adesogan and Dahl 2020; FAO 2023).

Smallholder farmers, who typically keep two dairy cows or buffalo per farm, are the primary source of revenue for the dairy industry in Asian and African countries (Kariuki et al. 2017). Millions of rural, peri-urban, and urban farmers in Sub-Saharan Africa depend heavily on producing dairy products for their livelihoods (Bateki et al. 2020). In 2019, at least 30% of the global milk production was done i developing and emerging countries like India, Brazil, and Pakistan (Ramírez-Rivera et al. 2019; FAO 2021). A growing market opens new opportunities in the dairy value chain and forces smallholder farmers to adapt and stay competitive in a constantly changing business environment (Chindime et al. 2016). Hence, effectively managing dairy farms is of the utmost significance to guarantee productivity and economic feasibility. One potential strategy for improving the efficiency and productivity of milk supply in developing countries is integrating a dairy recording system (Limo 2017). Dairy recording involves systematically collecting and evaluating data concerning milk production, quality, and various performance parameters of dairy cows. ICAR (2022a) described that the milk recording process commences by gathering pertinent information, such as animal identity, the calving date of milking cows, the quantity of milk produced, and the specific date and time or period during which milking occurs. The practice of milk recording enables farmers to monitor the productivity of individual cows and the overall herd, which helps as a valuable resource for informing managerial decisions (Balaine et al. 2020). According to Kamphuis et al. (2015), using data obtained through milk recording is of immense value in aiding farmers in making informed decisions on aspects such as feeding practices, reproductive strategies, and culling procedures. Dairy farm records are also very useful for making financial planning, helping the government with administration and extension work, and analysing the dairy sector as a whole (Yadeta et al. 2020).

Developed countries tend to have centralized systems in which a central authority or organization predominantly oversees data collecting, processing, and maintenance. In contrast, decentralized recording systems are more prevalent in developing nations (Kosgey et al. 2011; Limo 2017). Opoola et al. (2019) explained that rising demand for animal products and increased export potential have prompted private or public stakeholders in the dairy industry to begin building record-keeping systems in developing countries. However, the implementation of dairy recording in developing countries is generally deficient. According to Yadeta et al. (2020), conventional practices and daily routines of dairy farmers in developing countries may make setting up comprehensive record-keeping methods difficult. Therefore, this study aims to identify record-keeping tools for smallholder dairy farmers in developing countries to gain an overview of their development and provide comprehensive input on potential challenges and insights for future improvements. In addition to the technical aspects of recording, along with the increasing development of digital technology, the study also provided an overview of technology utilization in record-keeping tools in developing countries over the past decade. The feedback from this study will be useful to various stakeholders, including farmers, dairy cooperatives, dairy processing companies, veterinarians, the government, IT industries, and others. This input will help enhance the functionality and features of the tool being developed, thereby contributing to the advancement of technology. According to ICAR, recording will not only provide benefits to farmers for their daily management decisions, but it will also help other stakeholders with strategic solutions for sustainable dairy cattle management, such as herd development, selection decisions for breeding, or international trade (ICAR 2022b).

## Methodology

The study adapted a checklist from the Preferred Reporting Items for Systematic Reviews and Meta-Analyses (PRISMA) framework described by Page et al. (2021). Referring to the PRISMA outline, the paper selection methodology carried out in this study was divided into three stages, namely identifying strategies and selection criteria for the required publications, conducting a thorough selection process to assess and evaluate the nominated articles, and analysing the gathered information to present the resulting findings.

### Identification strategy and selection criteria

At the beginning, the search for scientific articles was conducted on the following databases: Web of Sciences, Scopus, Science Direct, and Springer. In addition, so-called “grey literature” (theses, reports, manuals) from Google Scholar was added. The article search used Boolean operators (AND and OR), and the syntax of the keyword string used was (“recording” OR “record-keeping”) AND (“dairy” OR “cow” OR “milk”) AND (“smallholder dairy farm*” OR “developing countr*”). Following this search, additional studies were identified by reviewing the reference lists in the selected publications. The range of publication years used in this study was from 2013 to 2023, and only publications written in English were considered. The year’s selection was derived due to the limited number of publications related to the topic over the previous five years.

### Selection process

**Fig. 1:**
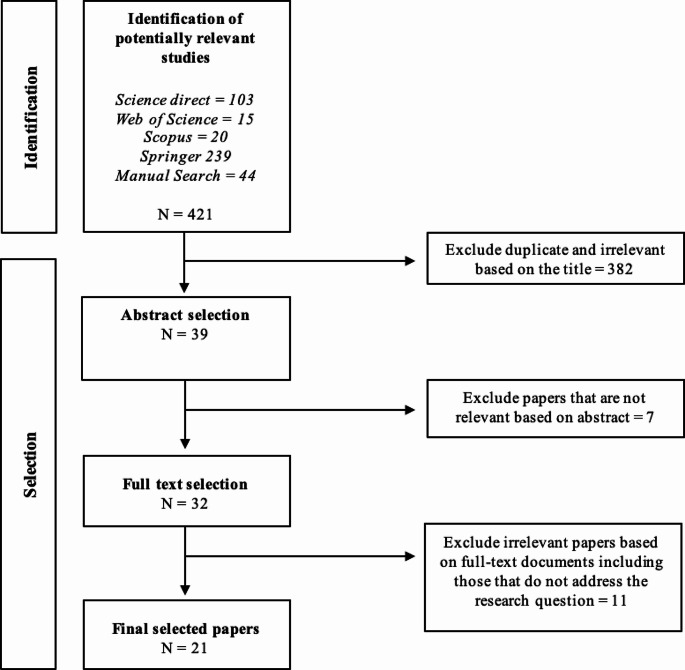
Identification and selection process of papers modified from Booth et al. (2016)

In the next step, a data extraction form was devised to systematically collect and retrieve all the requisite information for the study. Figure 1 illustrates the identification and selection process. The provided information was documented in an Excel sheet and consisted of the study’s title, authors, abstracts, year of publication, and study location. At this step, the presence of duplicate papers was also assessed. The first author carefully read the entire article for a more thorough evaluation. Furthermore, comprehensive information precisely needed to respond to the research inquiries was included in the worksheet. The selected papers aimed to answer the following queries: In which developing countries have dairy recording tools been developed? What types of recording tools have been developed? What core components of dairy recording systems have been invented? Which stakeholders have been involved in the design of dairy recording tools? What are the digital technologies that have been developed?

### Data analysis

We identified two articles that discussed more than one recording tool. Therefore, 24 different dairy recording tools were included in the analysis. The dairy recording tools were classified into four core components defined by FAO (2016): animal identification and registration (I&R), animal traceability (AT), animal health information (AHI), and animal performance recording (PR). Additionally, dairy recording tools in this study were categorised into five information technology (IT) categories derived from Shambhavi et al. (2023). It encompasses mobile phone applications, desktop applications, web applications, networks, and the Internet of Things (IoT). Then, the stakeholders contributing to the development of the tools were identified. In addition, obstacles and challenges to the implementation of digital recording tools were analyzed.

## Results and discussion

### Geographical breakdown of selected papers

**Fig. 2:**
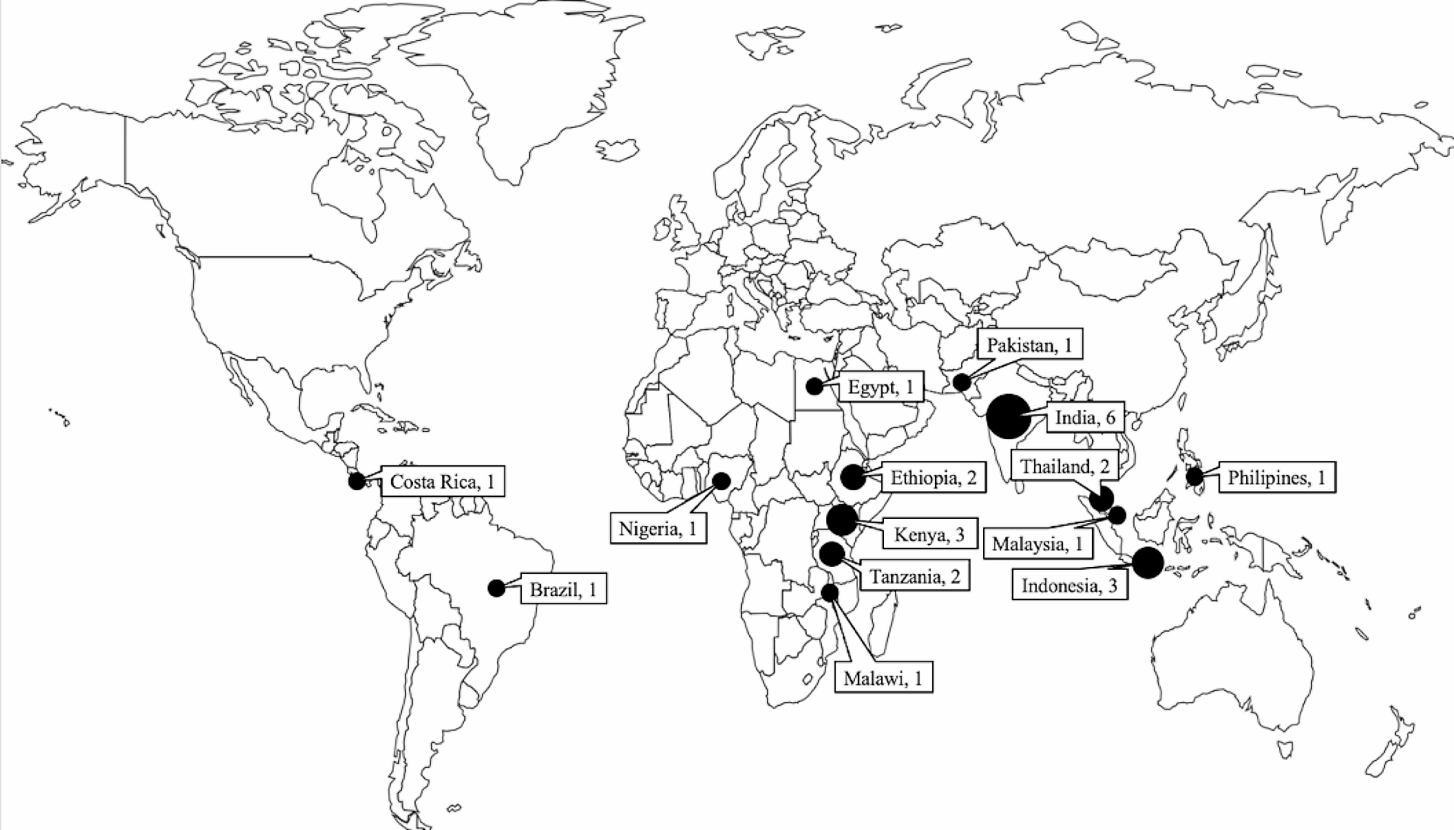
Distribution of shortlisted tools by country

Diverse stakeholders from academia, the government, farmer cooperatives, and the private sector have contributed to developing these record-keeping tools, which are found in 14 developing countries (Fig. 2). Some recording tools are even available in more than one country.

The selected papers indicate the development of recording tools in Asia, Africa, and Latin America. India is the country where most recording tools were designed, followed by Kenya and Indonesia. The reason for this expansion in India is likely connected to its position as one of the leading milk producers in the world, which necessitates enhancing its dairy industry. According to the Organisation for Economic Co-operation and Development (OECD) and Food and Agriculture Organisation (FAO), it is projected that India and Pakistan will collectively contribute to over half of the worldwide surge in milk production over the next ten years (OECD-FAO 2020). It signifies the notable influence of India’s emergence as the leading global milk producer, with a production output of 192 million metric tonnes per year in 2019.

### Current recording tools and their practices in digital technology

The findings indicate that the dairy recording tools in the selected studies were predominantly designed according to the four core components outlined by FAO (2016), as presented in Table 1. Animal identification and registration (I&R) refers to the registration of animals, including details about the animal’s uniqueness and background, such as birth, parentage, sex, genetic information, breed, and pedigree (Reinemann 2019). Animal traceability (AT) lays the groundwork for observing and policing hygienic standards and ensuring the security of food items of animal origin. FAO (2016) also added that AT is concerned with the assurance of food safety and quality control, which involves the identification, tracking, and regulation of animal movements, as well as animal health inspection and certification. Data analysis of this component is also used to trace animal history, particularly for disease control and food safety. It is also related to animal health information (AHI). Data collection in the AHI component included gathering information on diseases, vaccinations, treatments, and overall health status. The other component, animal performance recording (PR), encompasses the collection of data pertaining to several aspects of animal performance, including, but not limited to, growth rate, milk production, and reproductive efficiency. Balaine et al. (2020) stated that this information is essential for improving profitability and ensuring the long-term viability of dairy businesses.

**Table 1:** Recording tools introduced based on the core components and IT categories in the shortlisted articles

No	Recording tools	I&*R*	AT	AHI	PR	IT Type	Authors
1	REKS-EL version 1.0	√	-	√	√	Mobile Phone Application	(Desviani et al. 2022)
2	Dairy Kannada app	√	-	√	√		(Satyanarayan et al. 2018)
3	SMAFRS application	√	-	-	√		(Fouad et al. 2021)
4	Cowlog	√	-	-	√		(Phisanbut et al. 2021)
5	System for cattle disease diagnosis	√	-	√	-		(Anggraeni et al. 2013)
6	Vet Africa	√	√	√	-	Combination of mobile phone and web applications	(Beyene et al. 2018)
7	D`Cattle IS / SI-Lemper	√	-	√	√		(Ardjo et al. 2017)
8	Fodjan	√	-	-	√		(Bateki et al. 2021)
9	TANLITS (Tanzania national livestock identification and traceability system)	√	√	√	-		(George et al. 2021a)
10	Herdman	√	-	√	√	Combination of mobile phone application with IoT	(Daum et al. 2022)
11	System for heat detection	√	-	-	√		(Arago et al. 2022)
12	iCow	√	-	√	√		(Daum et al. 2022)
13	Automatic milk weighing scale	√	-	-	√		(Kaunkid et al. 2022)
14	Thermometer RFID	√	-	√	-		(Debnath et al. 2017)
15	IoT Smart based	√	-	-	√		(Mateen et al. 2018)
16	MATs (The Malaysia Animal Traceabillity System): ePermit1, eQuarantine,	√	√	√	-	Combination of IoT and web application	(Salina and Azmie 2013)
17	MooCare model	√	-	-	√		(Righi et al. 2020)
18	Cattle recognition system	√	-	-	-	Combination of IoT and network	(Kumar et al. 2017)
19	mSMSRS	√	-	√	√	Network	(Chiumia et al. 2020)
20	MPL datasheets	√	-	-	√	Desktop Application	(Migose et al. 2020)
21	The VAMPP Bovine management information system	√	-	√	√		(Sánchez et al. 2020)
22	Farm tree	√	-	√	√		(Daum et al. 2022)
23	Digital dairy farm management system	√	-	√	√	Web Application	(Parmar and Patel 2016)
24	Manual recording	√	-	√	√	No IT implemented	(Desviani et al. 2022)

* Animal identification and registration (I&R), animal traceability (AT), animal health information (AHI), animal performance recording (PR)

The recording may be preserved using various techniques, from manual record-keeping to digitization. Manual records usually include card files, notebooks, cow charts, labels, and colour coding that are handwritten while digitization through computers and mobile devices (Yadeta et al. 2020). Different information technologies were used (Table 1) during the development of milk recording tools, such as mobile phones, desktops, websites, networks, and IoT applications. The findings show that 15 out of 24 reported tools use mobile phone applications.

The extensive utilisation of mobile phones and rapid changes in information and communication technology (ICT) offer new possibilities for innovative methods of collecting data. The widespread use of mobile phone applications as recording tools in dairy farming is particularly noteworthy due to the convenience and simplicity it has. Rotondi et al. (2020) added that mobile phones can facilitate cost-effective and efficient communication, particularly in regions with inadequate infrastructure in sub-Saharan Africa and South Asia. Furthermore, other findings from this study indicate that apart from utilizing mobile phone applications, the IoT is also considered a potential alternative for recording tool support. At least 9 of the identified recording tools take advantage of IoT. The findings discovered that IoT is frequently integrated with several IT types, such as networks, mobile phones, and web applications, to enhance the efficiency and user-friendliness of the recorded technologies. In line with the statement of Kaunkid et al. (2022), the IoT can automate processes, improve overall effectiveness in dairy farm operations, and provide real-time data as one of the influencing factors for farm evaluation. The IoT enables farmers to get current data regarding the performance of their cattle, empowering them to make prompt decisions to enhance efficiency and output.

#### Animal identification and registration (I&R)

All the chosen studies have incorporated I&R components throughout the recording tools setup (Table 1). The results show that the developers acknowledge the significance of incorporating this component as a crucial aspect in creating a recording tool. This corresponds to what was set by ICAR (2022a), establishing an official recording system requires utilising an animal identification method that is easily distinguishable and exclusive for each animal. ICAR (2018) also specified that animal identification must be distinctive, formally recorded, and never duplicated. It can be done by labelling and tracking keeper-owners and animals through a tag, tattoo, brand, or electronic devices. As an example, the studies of Salina and Azmie (2013); Ardjo et al. (2017); Satyanarayan et al. (2018); Fouad et al. (2021); Daum et al. (2022) have applied ear tags integrated with barcodes which are embedded with radio-frequency identification (RFID), which can be linked to mobile devices, to access information related to the animals, involving the country of origin, pedigree, date of birth, sex, etc. Qiao et al. (2019) and Shen et al. (2020) found that RFID ear tags and visual tags are the best ways to identify individual cattle.

#### Animal traceability (AT)

Only three of the selected articles reported dairy recording systems associated with the traceability of life animals.

According to Opoola et al. (2019) and Yadeta et al. (2020), a number of factors contribute to this condition; for example, insufficient infrastructure, inadequate resource allocation, and limited regulatory support have all made it difficult for developing countries to integrate recording tools. The countries implementing AT in this study consist of Malaysia, Ethiopia, and Tanzania in which numerous stakeholders participate in a centralised government system. The research identified that developed animal traceability recording tools have provided features including livestock identification, quarantine, animal movement and disease information to detect and mitigate disease transmission. Typically, data is gathered through field investigations and subsequently forwarded to higher authorities. The data that has been collected is recorded on paper-based or in a digital format. The evaluated data will eventually be communicated to the responsible officer, who, if a suspicious case is identified, such as a case of certain diseases, will dispatch an officer to the farm for an investigation and report the details to the disease incident report system. As defined by Salina and Azmie (2013); George et al. (2021b), implementing a tracking system is typically conducted on a broad scale under the supervision of authorised personnel at the national level, such as those within the animal health department of the nation to which it applies. Nevertheless, constructing and sustaining a traceability system can be costly since developing countries frequently need new infrastructure, including reliable internet connectivity, databases, and digital technology. Yet, the importance of traceability systems is increasing due to the growing consumer demand for secure and nutritious animal-derived food products (Mutua et al. 2018).

#### Animal health information (AHI)

The study found that vaccinations and diseases were the most recorded health information. The data captured includes the type of vaccine provided, dosage, date of treatment, and the animal’s physical condition by collecting information on body temperature, heart rate, and other clinical signs. Animal health officers assigned to check directly in the field typically record this information by observing the indicated cattle for clinical symptoms (ICAR 2022c). The findings of this observation are generally reported by making use of mobile phone apps that provide diagnostic results, allowing farmers to afford further treatment quickly. The usage of rectal temperature embedded with RFID (Debnath et al. 2017) and the development of sensors to monitor cattle activities using Internet of Things (IoT) technology that is connected to computers or smartphones (Mateen et al. 2018) are some other recording devices that have been developed for health recording. Other recording tools, such as Herdman (Daum et al. 2022), enable farmers to receive feedback on the health status of their cattle based on information entered by field officers on their mobile phones.

#### Animal performance recording (PR)

**Fig. 3:**
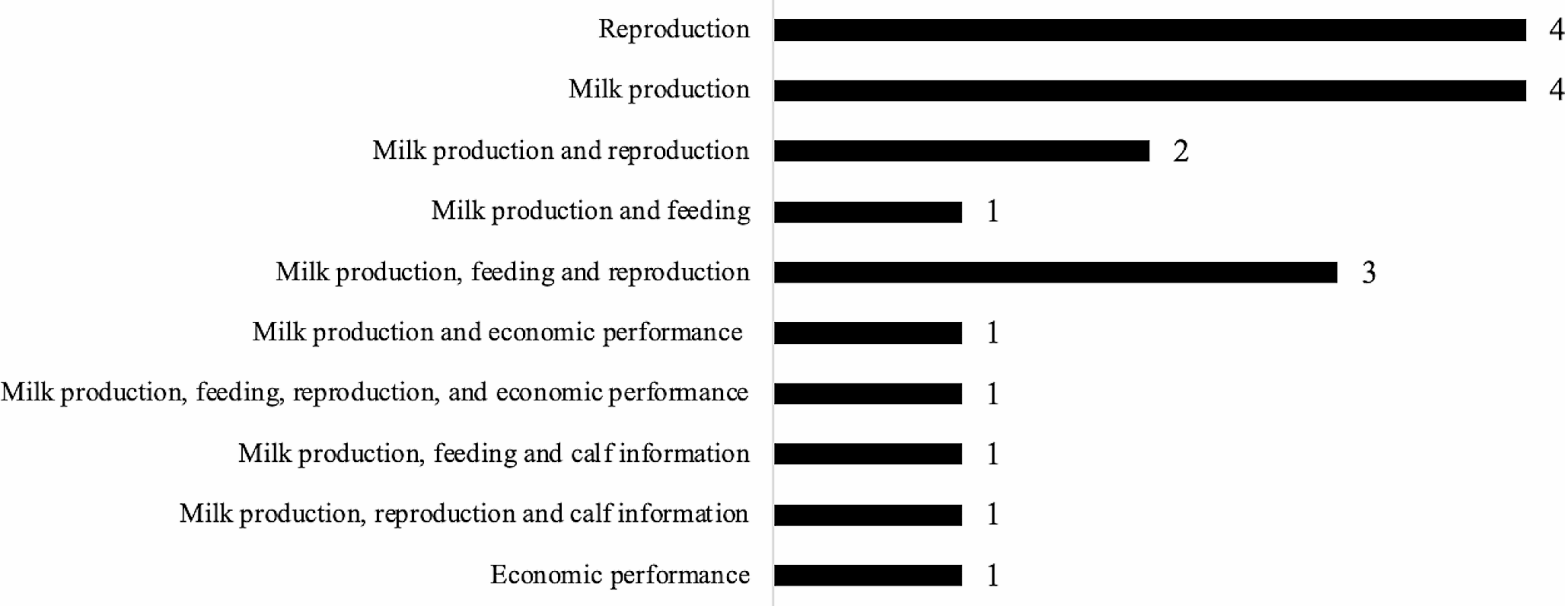
Number of animal performance tools reported by type

Milk recording tools were the most developed PR component, as presented in Fig. 3. In addition to milk production, reproduction and feeding were also widely developed as PR indicators in the selected articles. Nevertheless, the specific production data gathered in the selected papers remains inaccessible. There is no clear information about whether the data was collected on individual animals or the entire herd of cattle (e.g., milk yield per cow, feed intake per cow, etc.).

Despite the utilisation of digital technologies, such as mobile phones and PCs, for data storage, most production performance recordings in the selected study relied on manual input. The study found only one device that recorded individual milk production using automatic input. This recording tool has been designed by incorporating a weigh scale mechanism and an embedded system onto a wheelbarrow which is subsequently linked to a mobile device, enabling the real-time recording of milk production data for individual cows (Kaunkid et al. 2022). In most cases, farmers are able to enter data every day. Certain tools (e.g., Herdman, Farm Tree, iCow) offer technical information pertaining to feedback on reported data and alerts concerning the due dates of outstanding activities (e.g., A.I., pregnancy diagnosis, drying, calving date, and so forth) (Daum et al. 2022).

As previously described, we identified only one recording tool that provides individual performance in milk production (Kaunkid et al. 2022). Pereira et al. (2011) and Ask-Gullstrand et al. (2023) argued that milk production data allows the identification of animals for breeding replacement, contributes to genetic evaluation, and can even assist in pregnancy diagnosis. Accurate individual cow identification is needed to achieve accuracy and provide valuable information in dairy farming. Individual records can enhance farm management decisions at the individual cow and herd levels, aiding in genetic evaluation, selection, and management choices (Fu et al. 2022; Lassen et al. 2023). Farmers can optimize milk production by modifying each cow’s diet and nutrition using milk production data. Variations in milk production can indicate a cow’s reproductive health, enabling farmers to make informed decisions regarding heat cycles, pregnancy, and the management of breeding programs. Individual milk production data can also be used to identify high-performing cows with favourable genetic characteristics, which are essential for breeding strategies that enable farmers to choose cows with desirable traits. With accurate information, farmers can improve breeding decisions, boost herd performance, and ensure the health of their livestock. In addition, the gathering and assessment of production data are essential for improving profitability and ensuring the long-term viability of dairy businesses (Balaine et al. 2020).

### Stakeholder involvement in the recording tools development

**Fig. 4:**
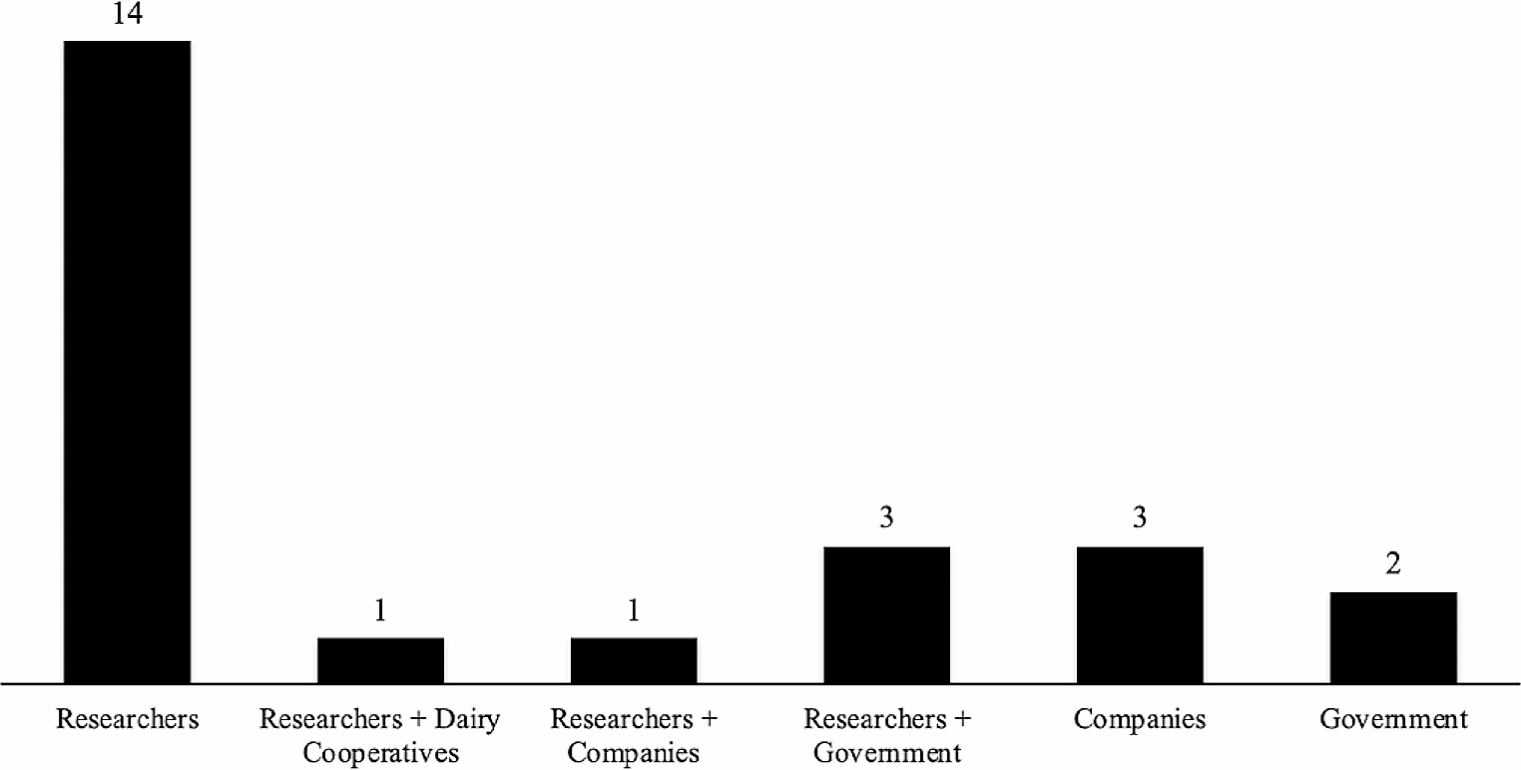
Stakeholders’ involvement in the recording tools of the shortlisted studies

Our study identified the involvement of four different stakeholders (researchers, government entities, farmers´ cooperatives, and companies) in developing recording tools. Figure 4 shows that researchers had greater activity in the development of recording tools compared to others, as they created a total of 14 various recording tools. However, the study reveals that most tools created by researchers are currently in prototype form and have not yet been fully implemented, which may require additional modifications before being utilised in real-life situations. Moreover, this study outlines that the primary beneficiaries of these tools, farmers and dairy cooperatives, are hardly engaged in the development of the devices. Only one partnership between researchers and farmers’ cooperatives was identified in the selected studies. As part of the actors in the dairy chains, engaging relevant stakeholders, especially farmers and dairy cooperatives, in the process of tool development should help improve sustainable recording for small-scale dairy farmers in developing countries. As stated by Triste et al. (2018), co-creation has the potential to establish a collaborative ecosystem wherein all participants are empowered to contribute to the development of more efficient and innovative solutions for a dairy farm. An example of a farmer-led initiative in the co-creation of recording tools is iCow, started by a farmer from Kenya, Su Kahumbu Stephanou, and supported by the US Department of State (https://icow.co.ke/icow-history/). This application was then further developed in collaboration with many parties, one of which is the African dairy genetic gains project. This project has been extended to multiple countries, including Kenya, Tanzania, Nigeria and Ethiopia, and involves the cooperation of farmers´ organizations, research institutes, the government, and private sector (Mwai et al. 2017; Mrode et al. 2023). AsGeorge et al. (2021a) implied, the private sector possesses more resource-based leverage than other key players, such as the public sector. The private sector enables faster and more effective technology transfer due to the availability of well-equipped and innovative resources. Studies identified that recording tools such as iCow, herdman and farm trees(Daum et al. 2022) developed by the industry tend to be more durable and acceptable to farmers. Therefore, the development of recording tools needs to consider effective collaboration with the industry to provide stronger interactions.

### Challenges and potential improvements in the advancement of digital dairy recording tools

Recording tools in developing countries have improved considerably over the past decade. Of the 24 available tools, 23 have been applied digitally through digital devices, although most data entry is still done manually. The findings (Table 1) show that mobile phone applications were most widely developed as dairy recording tools in the selected studies. However, the findings of the chosen study highlighted that the unreliable internet connection significantly hindered the process of collecting data in the fields. Unreliable internet connections in some areas delay accessibility during data entry. Dealing with unstable or inadequate networks may pose challenges to the collection and transmission of data in real time. Strategies such as enhanced investments in broadband infrastructure could broaden internet accessibility (Dhehibi et al. 2023; Valentín-Sívico et al. 2023). Additionally, in some selected studies by Bateki et al. (2021) and Desviani et al. (2022), it was found that farmers did not keep regular records, which resulted in incomplete data being submitted. Multiple factors contribute to the farmers’ inconsistency in record-keeping. Some farmers believe data entry will consume their time due to the numerous farm-related tasks needed. Moreover, recording is also hampered by the huge amount of information that must be entered. Inadequate literacy skills, insufficient encouragement, and a need for more awareness regarding the significance of recording constrain farmers’ recording activities (Djokoto 2012; Yadeta et al. 2020). Due to a lack of capacity to interpret and analyse the available data, some farmers assumed that record-keeping had little effect on their farm operations. Thus, farmers need to be trained on how important it is to keep accurate and organised records so that they can see how better managing their animals and farm businesses as a whole can help them. It is also important to consider the regular feedback provided to farmers regarding their recordings activities in order to promote continuous recording activities among farmers.

In terms of technical farm management aspects, the finding reveals that there are no tools in the selected study that effectively use all core components in one device. Most of the tools only cover two or three core components. The most frequently integrated core components are I&R, AHI, and PR. A shortage of these components will probably lead to incomplete evaluations and feedback that may result in inaccurate and unsatisfactory outcomes (Kang 2013; Miao et al. 2018). However, farmers can combine the use of two or more tools to supplement missing data. Additionally, the application of sensors incorporated into mobile applications to gather data can also be carried out as an initiative towards addressing data deficiency, which enables the distant and regular monitoring of individual animals (Siberski-Cooper and Koltes 2022). Enhancing the process of gathering specific information on dairy cows, such as through the incorporation of on-farm data streams and automated monitoring systems, can improve the thoroughness and effectiveness of data gathering for assessing milk production, reproductive performance, and overall farm management (Celozzi et al. 2020). Madouasse et al. (2010) and Sánchez et al. (2020) added that conducting individual data enables the selection of specific animals within a group and adopting different management techniques. This empowers farmers to make well-informed decisions swiftly, improves their herds’ management, and enhances dairy cows’ health and production. Qi et al. (2022) emphasised the need for digital technology for sustainable development and higher production in dairy farming. Digital technology has the capability to speed up production processes, simplify operations, and gather data that aids farmers in decision-making and enhances management effectiveness.

Furthermore, establishing and maintaining the essential technology infrastructure for dairy recording necessitates investments in physical structures, machinery, and utilities. Thus, it is important to acknowledge the financial implications of the adoption of dairy recording tools, especially in developing countries. Sánchez et al. (2020) explored the expenses associated with providing computer systems, software licences, and other hardware necessary for data gathering and management can be relatively high. Despite this, Koltes et al. (2018) agreed that future advancements would necessitate real-time data collection, data management, and the creation of prediction models to facilitate early intervention to prevent or minimize losses in present and future animal populations. The initial investment required to procure dairy recording tools can be substantial, particularly for those with limited financial means. As defined by Mrode et al. (2020), the concern lies in producing simple, efficient, cost-effective, non-invasive, and long-lasting tools to collect accurate data on many aspects. The most effective solutions will enable frequent data capture and immediate transmission to adaptable databases for prompt user feedback. Therefore, the role of primary stakeholders, including researchers, farmers, and related industries, is needed to overcome these technical and non-technical obstacles collaboratively and individually by sharing knowledge, distributing resources, and employing expertise to manage these challenges successfully (Ramirez et al. 2019; Perry et al. 2022).

## Conclusions

The recording devices in the selected studies have been developed using different types of technologies integrated into mobile phones, websites, desktops, networks, and IoT applications. Identification of the livestock, tracking of the animals, health data, and livestock performance are among the recorded components. Only three recording tools are related to livestock tracking information, while milk production is the most developed recording component in the selected studies. Most recording tools are set on a digital device, primarily concentrating on production performance, specifically milk production, by integrating mobile phone applications with the Internet of Things (IoT).

Since practically everyone owns a mobile phone, using one is still a desirable option as a recording device. Conversely, the availability of supporting facilities such as internet access and ongoing record-keeping must be considered, particularly regarding routine and continuous data entry. It is also necessary to underline the importance of awareness of the benefits of record-keeping, for example, by providing farmers with training and regular feedback on the data entered. Agricultural advisory services play an important role in the dissemination of knowledge. The various stakeholders should, therefore, be involved in the further development of the recording tools.

## Data Availability

Not applicable.
